# Molecular mechanism of ethylene stimulation of latex yield in rubber tree (*Hevea brasiliensis*) revealed by *de novo* sequencing and transcriptome analysis

**DOI:** 10.1186/s12864-016-2587-4

**Published:** 2016-03-24

**Authors:** Jin-Ping Liu, Yu-Fen Zhuang, Xiu-Li Guo, Yi-Jian Li

**Affiliations:** Hainan Key Laboratory for Sustainable Utilization of Tropical Bioresources, College of Agronomy, Hainan University, Haikou, Hainan Province 570228 P. R. China; Service Center of Science and Technology, Rubber Research Institute, Chinese Academy of Tropical Agricultural Sciences, Danzhou, Hainan Province 571737 P. R. China

**Keywords:** Rubber tree, *Hevea brasiliensis*, Ethephon treatment, Ethylene stimulation, Rubber production

## Abstract

**Background:**

Rubber tree (*Hevea brasiliensis*) is an important industrial crop cultivated in tropical areas for natural rubber production. Treatment of the bark of rubber trees with ehephon (an ethylene releaser) has been a routine measure to increase latex yield, but the molecular mechanism behind the stimulation of rubber production by ethylene still remains a puzzle. Deciphering the enigma is of great importance for improvement of rubber tree for high yield.

**Results:**

*De novo* sequencing and assembly of the bark transciptomes of *Hevea brasiliensis* induced with ethephon for 8 h (E8) and 24 h (E24) were performed. 51,965,770, 52,303,714 and 53,177,976 high-quality clean reads from E8, E24 and C (control) samples were assembled into 81,335, 80,048 and 80,800 unigenes respectively, with a total of 84,425 unigenes and an average length of 1,101 bp generated. 10,216 and 9,374 differentially expressed genes (DEGs) in E8 and E24 compared with C were respectively detected. The expression of several enzymes in crucial points of regulation in glycolysis were up-regulated and DEGs were not significantly enriched in isopentenyl diphosphate (IPP) biosynthesis pathway. In addition, up-regulated genes of great regulatory importance in carbon fixation (Calvin cycle) were identified.

**Conclusions:**

The rapid acceleration of glycolytic pathway supplying precursors for the biosynthesis of IPP and natural rubber, instead of rubber biosynthesis per se, may be responsible for ethylene stimulation of latex yield in rubber tree. The elevated rate of flux throughout the Calvin cycle may account for some durability of ethylene-induced stimulation. Our finding lays the foundations for molecular diagnostic and genetic engineering for high-yielding improvement of rubber tree.

**Electronic supplementary material:**

The online version of this article (doi:10.1186/s12864-016-2587-4) contains supplementary material, which is available to authorized users.

## Background

Rubber tree (*Hevea brasiliensis* (Willd. ex A. Juss.) Müll. Arg.), cultivated in many countries of tropical area, is the primary commercial source of natural rubber. Natural rubber (cis-1,4-polyisoprene) produced from rubber tree has many outstanding performance properties and is an important industrial material which cannot be reproduced by synthetic alternatives [1, 2].

Natural rubber is synthesized through the mevalonic acid (MVA) pathway with isopentenyl pyrophosphate (IPP) as the substrate and building block [1–5]. But there were evidences that the possibility that 1-dexoxy-D-xylulose 5-phosphate/2-C-methyl-D-erythritol 4-phosphate (DEX/MEP) pathway is involved in rubber biosynthesis cannot be excluded [6–9]. Whatever the isoprenoid biosynthesis pathway, sucrose is the only precursor of natural rubber [8, 10].

Natural rubber biosynthesis takes place in latex and the latex is the cytoplasm of laticifer cells (the highly specialized cells in phloem). The latex harvesting is conducted by tapping or cutting the bark of rubber tree at regular intervals ranging from 2 to 5 days. Since ethephon (chloro-2-ethylphosphonic acid, an ethylene generator or releaser) was known to enhance rubber production, stimulation with ethephon has become a common practice in worldwide rubber tree plantations [11, 12]. Usually, a 1.5- to 2-fold increase of latex yield can be achieved by the treatment with ethephon [13, 14].

Much effort has been devoted to the elucidation of the mechanism of ethylene action. Stimulation with ethephon was shown to prolong the flow of latex after tapping [13, 15]. Two ethylene-responsive aquaporin genes (*HbPIP2;1* and *HbTIP1;1*) had been characterized and their functions possibly favor the prolongation of latex flow through regulation of water exchanges between inner liber and latex cells [16].

Physiological and biochemical studies showed that ethylene activated the general metabolism for latex regeneration between tappings, with adenylic pool, polysomes and rRNA contents accumulated in laticifers, and the activities of glutamine synthetase (GS) and chitinase up-regulated [13, 14, 17, 18]. Specifically, Tupy [19–21] showed that bark application of auxins and ethylene enhanced the invertase activity and sucrose utilization in latex by increase of latex pH. Cytosolic alkalinization might be explained by the early activation of H^+^-translocating ATPase by ethylene [22].

In view of the fact that the laticifer is a strong sink for sucrose and sucrose importation into laticfer and quebrachitol absorption may be important for sustained sucrose demands for latex regeneration, Dusotoit-Coucaud et al. [23–25] and Tang et al. [26] characterized several sucrose transporters and a polyol transporter, and suggested that they might be involved in ethylene-induced stimulation of latex production.

Liu et al. [27] constructed and screened two ethephon-induced latex SSH cDNA libraries, and found that the cDNAs associated with sucrose metabolism, regulation of coagulation, stability of lutoids and signal transduction were up-regulated and might be related to the ethephon action.

Due to the complexity of ethylene stimulation and the limitations of the methodologies, many researches have been undertaken on understanding the mechanism of ethylene action but it has still not resolved [28]. In contrast with the conventional methods such as single gene cloning and DNA microarrays which yield a limited amount of genetic information, RNA sequencing is powerful tool for analyzing differential gene expression with high resolution on the whole genome level [29, 30]. Particularly, transcriptome analysis can be employed to reveal relationships between plant gene expression and phenotypes [31–33]. Transcriptome sequencing technology has been applied to investigate the biology of rubber tree for generation of tissue-specific transcriptomal data [34] and genome sequence [35], development of molecular markers [36–39], identification of novel microRNAs [40], specific genes and gene families [41–43].

In the present study, the first RNA sequencing project for deciphering the molecular mechanism of ethylene stimulation of latex yield in rubber tree was performed. Our objective was to identify the relevant metabolic pathways or major ethylene-responsive genes. It should be noted that, we treated the rubber tree bark with ethephon for 8 and 24 h, considering that it should be an effect of relatively long duration and the response to the stimulation should at last be transmitted to certain metabolic pathways.

## Results and discussion

### Sequencing and de novo assembly

**Fig. 1:**
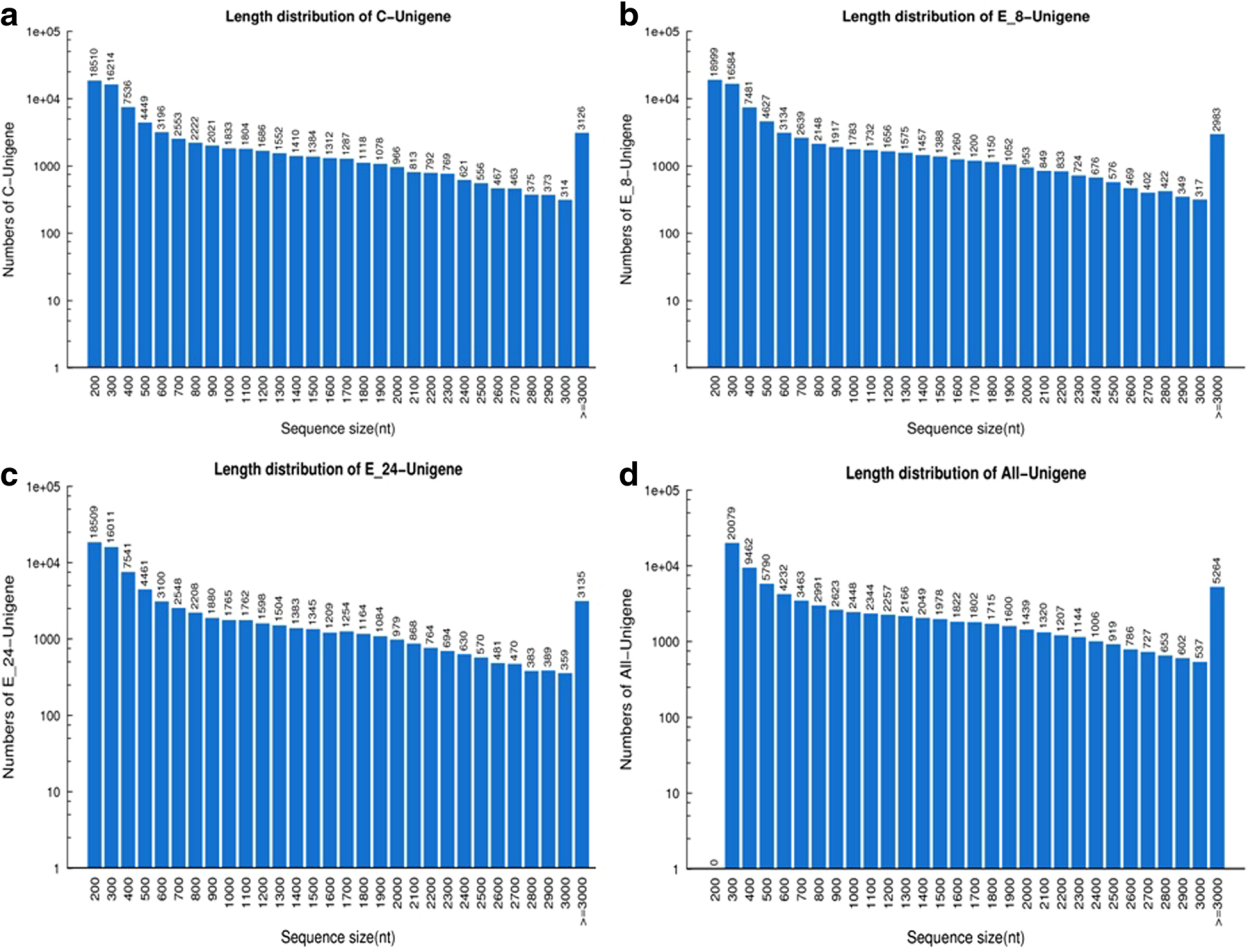
The length distribution of the unigenes. The length distribution of the unigenes of C sample (**a**), E8 sample (**b**), E24 sample (**c**) and the all-unigenes (**d**)

Three cDNA libraries from bark tissue, C (control), E8 (ethephon treatment for 8 h) and E24 (ethephon treatment for 24 h), were sequenced by Illumina deep-sequencing and a total of approximately 55, 54 and 54 million raw reads for C, E8 and E24 were generated, respectively. After removal of low-quality reads, adaptor sequences and ambiguous reads, about 53, 51 and 52 million high-quality clean reads for C, E8 and E24 were obtained, respectively, with the Q20 (percentage of sequences with sequencing error rate lower than 1 %) over 98 % for the three samples (Table 1). Assembly of all trimmed reads produced 138,182-140,407 bp contigs from the three libraries with the average length exceeding 350 bp. The contigs were joined into unigenes based on the paired-end information, generating 80,800 (C), 81,335 (E8) and 80,048 (E20) unigenes, with an average length of 1,101 bp and N50 of 1,875 bp (50 % of the assembled bases were incorporated into unigenes of 1875 nt or longer) (Table 1). All unigenes were longer than 200 bp, the length of 29,541 (34.99 %) unigenes ranged from 201 to 400 bp, and unigenes with length longer than 2000 bp accounted for 16.78 % (14,165) of total unigenes. The length distribution of the unigenes is shown in Fig. 1.

**Table 1:** Overview of the sequencing and assembly

	C	E-8	E-24	Total
Total Raw Reads	55,532,586	54,200,296	54,549,846	
Total Clean Reads	53,177,976	51,965,770	52,303,714	
Total Clean Nucleotides (nt)	4,786,017,840	4,676,919,300	4,707,334,260	
Q20 percentage	98.24 %	98.30 %	98.30 %	
N percentage	0.00 %	0.00 %	0.00 %	
GC percentage	43.33 %	43.48 %	43.31 %	
Contig				
Total Number	139,806	140,407	138,182	
Total Length (nt)	49,459,322	49,507,343	49,415,302	
Mean Length (nt)	354	353	358	
N50	667	659	683	
Unigene				
Total Number	80,800	81,335	80,048	84,425
Total Length (nt)	65,946,686	65,357,057	65,398,163	92,963,404
Mean Length (nt)	816	804	817	1101
N50	1,652	1,640	1,673	1,875
Total Consensus Sequences	80,800	81,335	80,048	84,425
Distinct Clusters	30,702	29,583	30,307	40,642
Distinct Singletons	50,098	51,752	49,741	43,783

### Functional annotation and classification

Function annotation of the generated unigenes was performed by searching the reference sequences using BLASTX against NT (non-redundant NCBI nucleotide database), NCBI’s non-redundant protein databases (NR), SwissProt, Kyoto Encyclopedia of Genes and Genomes (KEGG) and COG (Cluster of Orthologous Groups). A total of 59,452 significant BLAST hits (70.42 % of all unigenes) were returned. Among them, 55,936 (66.26 %), 54,741 (64.84 %), 35,557 (42.12 %), 33,721 (39.94 %), 23,044 (27.30 %), 45,515 (53.91 %) unigenes were found in NR, NT, Swiss-Prot, KEGG, COG and GO database, respectively. Summary of functional annotations of unigenes by aligning to the Nr, Nt, Swiss-Prot, COG, GO and KEGG databases is shown in Additional file 1.

**Fig. 2:**
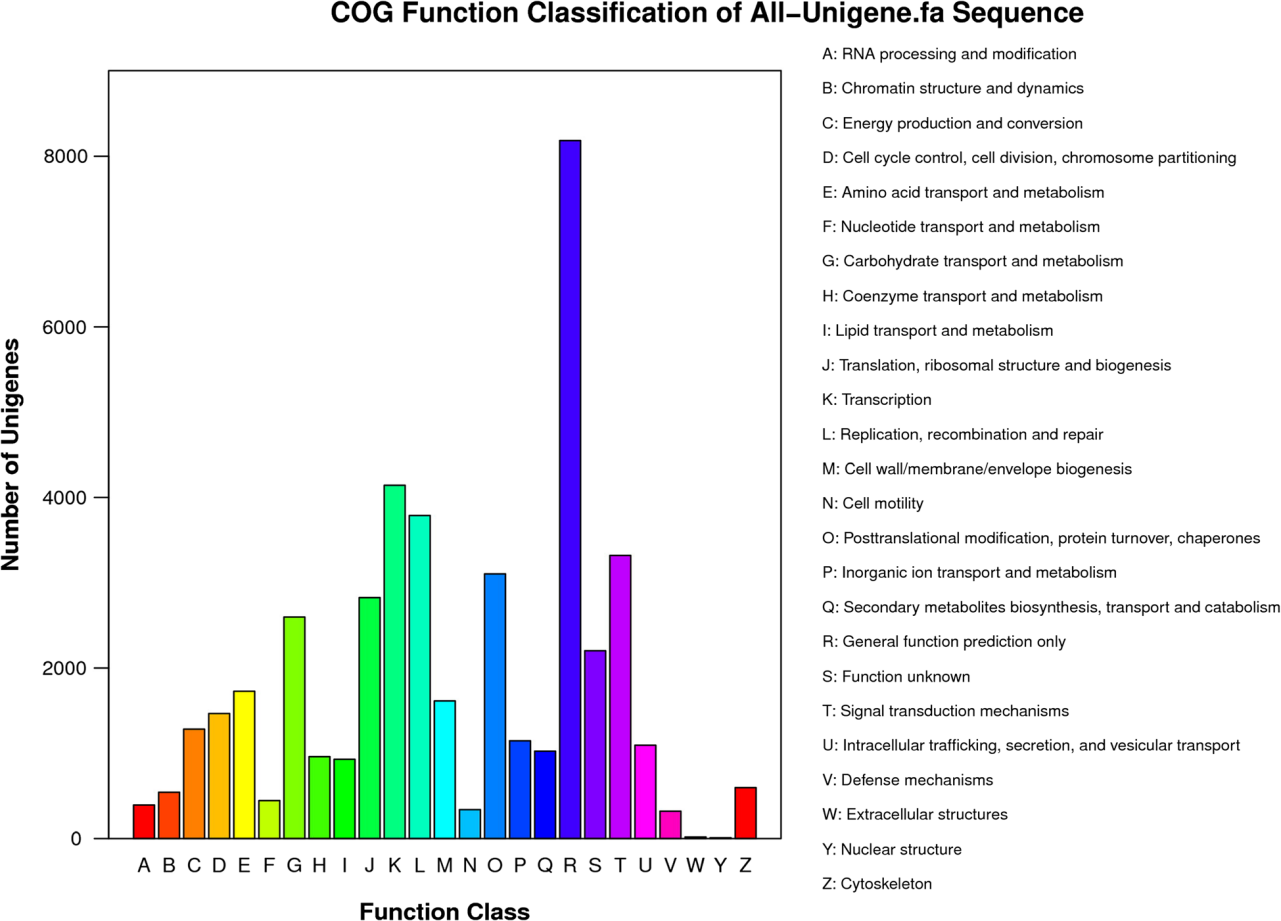
COG classification of the total assembled unigenes

COG classification revealed that 44,030 out of all the assembled unigenes were clustered into 25 functional categories (Fig. 2). The largest category was “General function prediction only” (8185, 18.59 %), followed by “Transcription” (4143, 9.41 %), “Replication, recombination and repair” (3787, 8.60 %), “Signal transduction mechanisms” (3319, 7.54 %) and “Posttranslational modification, protein turnover, chaperones” (3102, 7.05 %). “Nuclear structure” (9, 0.02 %) was the smallest group and 2202 (5.00 %) unigenes were annoted as “Function unknown”.

**Fig. 3:**
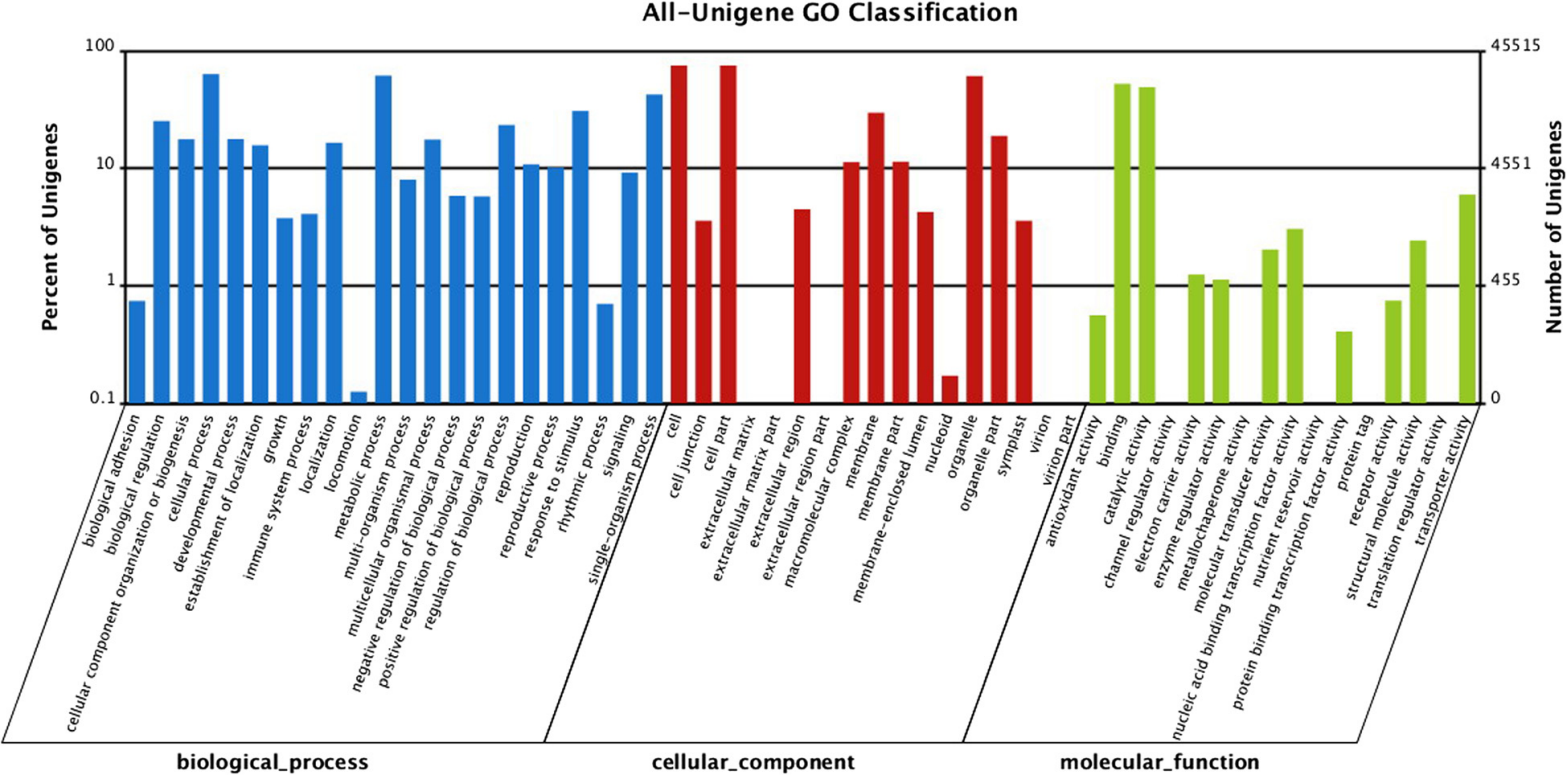
GO classification of the total assembled unigenes. The left y-axis indicates the percentage of genes for each functions; the right-axis indicates the correspondent number of genes

GO assignments were used to classify the predicted functions of the unigenes and total of 367,851 unigenes were classified into three main categories: biological process (178,004, 48.39 %), cellular components (135,748, 36.90 %) and molecular function (54,099, 14.71 %) (Fig. 3). In the “biological process” category, the top six largest categories were “cellular process” (28,776, 7.82 %), “metabolic process” (27,921, 7.59 %), “single-organism process” (19,263, 5.24 %), “response to stimulus” (14,053, 3.82 %), “biological regulation” (11,538, 3.14 %) and “regulation of biological process” (10,666, 2.90 %). As for the molecular function, unigenes with binding (23,778, 6.46 %), catalytic activity (22, 256, 6.05 %) formed the largest groups.

### Analysis of KEGG pathways and differentially expressed genes

KEGG pathway-based analysis was conducted to obtain a better understanding of the biological functions of the unigenes. The results showed that 33,721 annotated transcripts were mapped to 128 KEGG pathways. Of the 33,721 unigenes, 7470 (22.15 %), 3517 (10.43 %), 2289 (6.79 %) and 1717 (5.09 %) were involved in the metabolic pathways, the biosynthesis of secondary metabolites, the plant-pathogen interaction and the plant hormone signal transduction, respectively.

FPKM (Fragments Per Kb per Million reads) method was used to calculate the expression levels of the unigenes to identify differentially expressed genes (DEGs). A total of 5,326 up-regulated unigenes and 4,890 down-regulated unigenes were identified in E8 compared with C, and 4,440 and 4,934 unigenes were up-regulated and down-regulated in E24 compared with C. DEGs between C and E-8, and C and E-24 are shown in Additional file 2. The top 20 most up-regulated and down regulated genes between C and E-8, and C and E-24 are shown in Additional file 3.

By performing the KEGG pathway enrichment analysis, 23 and 17 significantly enriched metabolic pathways or signal transduction pathways of DEGs in E8 and E24 were identified, respectively (Additional file 4). To examine the molecular basis of the ethylene stimulation of latex yield in rubber tree, we concentrated on glycolysis, IPP biosynthesis pathway, C3 carbon fixation (Calvin cycle), plant hormone signal transduction and TFs in relation to rubber biosynthesis.

### DEGs involved in glycolysis and IPP pathway

**Fig. 4:**
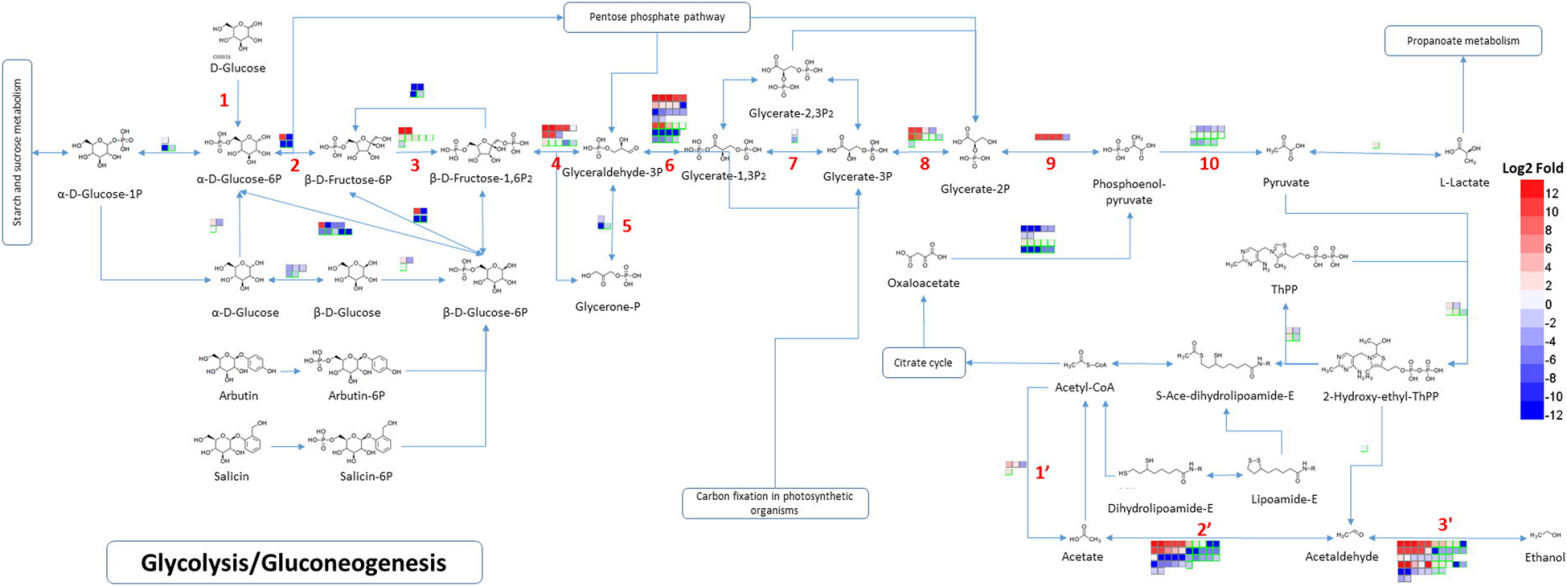
Differential expression of unigenes involved in glycolysis in E8 and E24 compared to C samples of *Hevea brasiliensis.* Glycolysis comprises 10 step reactions. In step 1, the enzyme hexokinase (HK) phosphorylates glucose by transferring a phosphate group from ATP to glucose forming glucose 6-phosphate. In step 2, glucose 6-phosphate is converted to its isomer fructose 6-phosphate by phosphoglucoisomerase (PGI). In step 3, the enzyme phosphofructokinase (PFK) catalyzes the conversion of fructose 6-phosphate into fructose 1,6-bisphosphate using another ATP to transfer a phosphate group to fructose 6-phosphate. In step 4, Fructose 1,6-bisphosphate aldolase (FBA) splits fructose 1,6-bisphosphate into dihydroxyacetone phosphate (DHAP, also glycerone phosphate) and glyceraldehyde 3-phosphate (G3P). In step 5, triose-phosphate isomerase (TPI or TIM) catalyzes the reversible interconversion of DHAP and G3P. In step 6, G3P is converted to 1,3-bisphosphoglycerate (1,3-BPG) by glyceraldehyde 3-phosphate dehydrogenase (GAPDH). In step 7, phosphoglycerate kinase (PGK) catalyzes the reversible transfer of the phosphate group from 1,3-BPG to ADP generating 3-phosphoglycerate (3-PG) and ATP. In step 8, the conversion of 3-PG to 2-phosphoglycerate (2-PG) is catalyzed by phosphoglycerate mutase (PGM). In step 9, enolase (or phosphopyruvate hydratase) catalyzes the dehydration of 2-PG to form phosphoenolpyruvate (PEP). In step 10, pyruvate kinase (PK) catalyzes the transfer of the phosphate group from PEP to ADP, yielding pyruvate and ATP. In an acetyl-CoA metabolic pathway, acetyl-CoA synthetase (ACS) (or acetate : CoA ligase) catalyzes the interconversion between acetyl-CoA and acetate (Step 1′), aldehyde dehydrogenase (ALDH) reversibly catalyze the conversion of acetate into acetaldehyde (Step 2′) and alcohol dehydrogenase (ADH) facilitates the interconversion between aldehydes and alcohols (Step 3′). Cells with gray border lines in the upper rows represent differentially expressed unigenes in E8 compared to C and cells with green border lines in the lower rows represent differentially expressed unigenes in E24 compared to C. Relative levels of expression are showed by a color gradient from low (*blue*) to high (*red*)

Glycolysis pathway is shown in Fig. 4. In the glycolysis pathway, the genes of PFK (in step 3), FBA (in step 4), PGM (in step 8) and PK (in step 10) were significantly up-regulated in both E8 and E24 compared to C, and the gene expression of GAPDH (in step 6) was up-regulated in E24 compared to C (Fig. 4, Additional file 5). It is well known that the PFK reaction is the first irreversible step committed to glycolysis and PK is responsible for producing pyruvate as the end product of the pathway. Both PK and PFK reactions are crucial points in regulating the rate of glycolysis and in other words these steps are rate-determining steps [44, 45]. In the “bottom up” regulatory mode of plant glycolysis, primary and secondary regulation are exerted at the levels of PEP and fructose 6-phosphate, respectively [44, 45]. In addition, transgenic study demonstrated that PGM also plays an important role in the control of glycolysis [46, 47].

Glycolysis pathway not only plays a crucial in energy generation for latex generation but also provides carbon building blocks for the biosynthesis of rubber and other organic constituents of latex [20, 44, 45]. Particularly, the breakdown of glucose by glycolysis produces the acetyl coenzyme A (acetyl-CoA) as the direct precursor in the MVP pathway, as well as the G3P and pyruvate which are the precursors in DEX/MEP pathway [48]. Up-regulation of the key enzyme genes described above possibly leads to an elevated rate of flux throughout the entire glycolytic pathway. Thus, the ethephon treatment enhances the latex yield through the rapid acceleration of the glycolysis for replenishing the precursors consumed in the biosynthesis of IPP and natural rubber molecules.

Moreover, we found that, in the metabolic pathway of acetyl-CoA into alcohol, the ACS (in step 1′) and ALDH (in step 2′) were down-regulated in both E8 and E24 compared to C (Fig. 4, Additional file 5). The down-regulation of the ACS and ALDH leads to the inhibition of the acetyl-CoA metabolic pathway under ethephon treatment, which may favor for the acetyl-CoA flowing toward the rubber synthesis.

In contrast, the IPP pathway was not found to be significantly enriched in this study and specifically, 3-hydroxy-3-metylglutaryl coenzyme A reductase (HMGR), which plays only limiting role in plant isoprenoid biosynthesis of MVA pathway [49], was slightly down-regulated (Additional files 6 and 7). This implied that carbohydrate utilization by glycolysis and availability of the precursors for IPP biosynthesis may critically determine the rate of rubber production, and the IPP pathway accounts little for the effect of ethephon stimulation. Although biosynthesis of natural rubber begins with the IPP pathway but the result that ethylene has little direct effect on accelerating rubber biosynthesis has been reported [28]. Interestingly, in one report about the mechanisms underlying super productivity of rubber tree, many rubber-biosynthesis-pathway genes showed no differential expressions between the super-high-yielding clone and the control [50].

### DEGs involved in carbon fixation

**Fig. 5:**
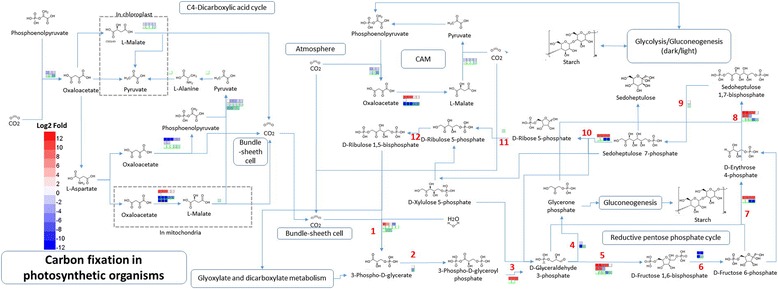
Differential expression of unigenes involved in carbon fixation in E8 and E24 compared to C samples of *Hevea brasiliensis.* The C3 carbon fixation proceeds through 13 steps in three phases. In carboxylation phase, ribulose-1,5-bisphosphate carboxylase/oxygenase (RubisCo) adds carbon dioxide to ribulose-1,5-bisphosphate (RuBP) to generate 3-PG (Step 1). Reduction phase include two reactions: PGK catalyses the phosphorylation of 3-PG by ATP to form 1,3-BPG (or 3-phospho-D-glyceroyl phosphate) and ADP (Step 2); GADPH catalyses the reduction of 1,3-BPG by NADPH to produce G3P (Step 3). The regeneration phase of the cycle comprises 9 steps and is initiated by the enzyme TPI which catalyses the interconversion of G3P and DHAP (Step 4). Then fructose-1,6-bisphosphate aldolase (FBA) reversibly catalyses the aldol condensation of G3P and DHAP into fructose-1,6-bisphosphate (Step 5). The resulting fructose-1,6-bisphosphatase is converted into fructose 6-phosphate by fructose-1,6-bisphosphatase (FBP) (Step 6). Transketolase (TK) catalyzes the transfer of a 2-carbon fragment from fructose 6-phosphate to G3P, affording erythrose-4-phosphate and xylulose-5-phosphate (Step 7). Erythrose-4-phosphate and a DHAP are converted into sedoheptulose-1,7-bisphosphate by aldolase (Step 8). The cleavage of sedoheptulose-1,7-bisphosphate into sedoheptulose-7-phosphate and an inorganic phosphate ion is catalyzed by sedoheptulose-1,7-bisphosphatase (SBP) (Step 9). The reversible interconversion of sedoheptulose-7-phosphate and G3P into ribose 5-phosphate and xylulose 5-phosphate is catalyzed by TK (Step 10). Ribose-5-phosphate isomerase (RPI) interconverts ribose-5-phosphate and ribulose-5-phosphate (Step 11). Finally, phosphoribulokinase (PRK) phosphorylates ribulose-5-phosphate into RuBP (Step 12). Cells with gray border lines in the upper rows represent differentially expressed unigenes in E8 compared to C and cells with green border lines in the lower rows represent differentially expressed unigenes in E24 compared to C. Relative levels of expression are showed by a color gradient from low (*blue*) to high (*red*)

The C3 carbon fixation (Calvin cycle) is demonstrated in Fig. 5. The expressions of RubisCo(in step 1), GADPH(in step 3), FBA(in step 5), aldolase(in step 8) and SBP(in step 9) were shown to be up-regulated in response to ethylene in both E8 and E24 samples (Fig. 5, Additional file 8). In the Calvin cycle, extensive studies demonstrated that RubisCO [51, 52], SBP [53–55] and aldolase [56, 57] dominate control of photosynthetic carbon fixation and they represent the rate-limiting enzymes in the Calvin cycle (reviewed by Raines [58] and Skitt et al. [59]). The up-regaulation of these enzymes in governing substrate flux through the Calvin cycle in response to the application of ethephon speeds up carbon fixation and further enhances the sustainable rubber productivity.

In plants, carbon-containing compounds including sucrose, IPP and natural rubber, ultimately comes from photosynthesis or from stored photosynthetic products. Wititsuwannakul [60] found that the HMGR activity in latex of rubber tree showed a diurnal variation pattern, and suggested that the regulation of the light-dark phase was probably due to physiological processes associated with photosynthesis and one or more of the three components of the rubber biosynthesis (acetyl-CoA, NADPH and ATP) become a limiting factor causing the decline in HMGR activity during the dark period. Therefore, to a certain extent, the photosynthetic carbon fixation may play an important role in ethylene-induced stimulation of latex production by constantly supplying carbohydrate for glycolytic transformation.

### DEGs of hormone signaling components and transcription factors

The responses of plant metabolism, growth, development and defense to exogenous ethylene application can hardly do without the ethylene perception and signal transduction [61]. Generally, hormone responses are the output of multiple pathway interaction and crosstalk [61–64]. Ethylene signaling may exert its functions via interaction with other major plant hormones such as brassinosteroid (BR), gibberellin (GA),auxin, cytokinin, salicylic acid (SA), jasmonate (JA), abscisic acid (ABA). The transcripts of signaling components such as ETHYLENE RESPONSE (ETR), ETHYLENE RESPONSE FACTOR 1 (ERF1), ETHYLENE INSENSITIVE 3 (EIN3) and EIN3 binding F-Box protein 1/2 (EBF1/2) for ethylene, BRASSINOSTEROID-INSENSITIVE 2 (BIN2), BRASSINAZOLE RESISTANT 1/2 (BZR1/2) and TCH4 for BR, DELLA for GA, INDOLE ACETIC ACID (IAA) for auxin, type-A response regulator (A-ARR) for cytokinin, TGA for SA, and MYC2 for JA were found to be remarkably accumulated after ethephon treatment (Additional files 9 and 10). Although all these hormones have been reported for being linked to growth regulation in certain manner [64], how the ethylene interacts and coordinates with other hormones in relation to the stimulation of latex production in rubber tree still remains to be elucidated.

Transcription factors (TFs) play important roles in the control of many of the biological processes in a cell or organism by the regulation of gene expression [65]. In addition, TFs also play crucial roles in the cross-talk between hormone signalling pathways [66]. A total of 1752 DEGs of putative TFs were identified in this study (Additional file 11) and the top five up-regulated and down-regulated TFs in E8 and E24 compared to C were listed in Additional file 12. Among them, the largest gene family was the The v-myb avian myeloblastosis viral oncogene homolog family (MYB) (88, 9.10 %), followed by MYB related (67, 7.44 %), the basic helix-loop-helix family (bHLH) (58, 6.44 %), C2H2 family (52, 5.77 %), and the ethylene-responsive element binding factor family (ERF) (44, 4.88 %). Although their functions relevant to ethylene responses for the production enhancement of rubber remained to be unknown but the data will be a valuable resource for the discovery of candidate genes related to the complex regulatory networks involved in the response.

**Fig. 6:**
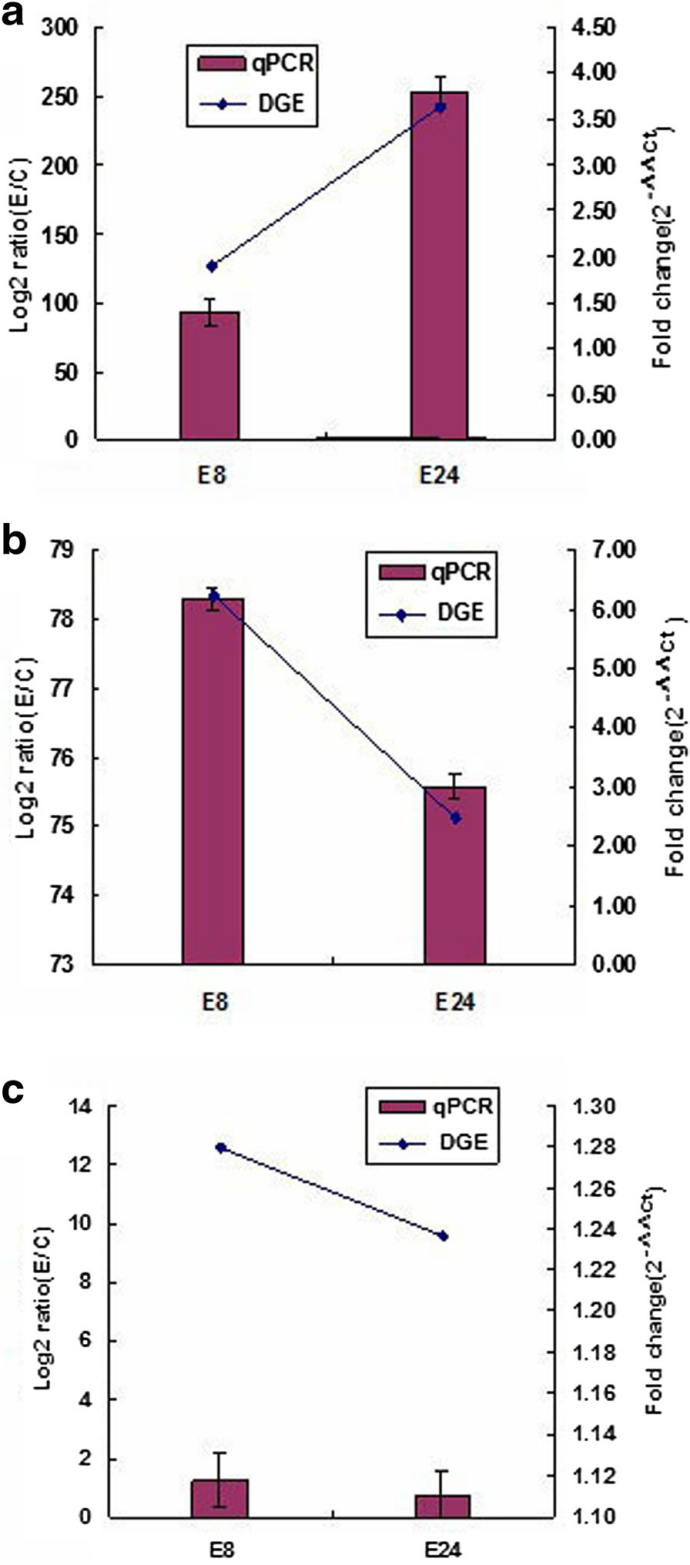
qRT-PCR validation of three DEGs involved in glycolysis and carbon fixation of *Hevea brasiliensis* bark. The axis indicates treatments; the y-axis indicates relative expression level. **a** *PFK*; **b** *Rubisco*; **c** *GADPH*

The results obtained by qRT-PCR analysis matched the expression found by transcriptome analysis (Fig. 6). The consistent expression patterns further increased the confidence of our findings and the transcriptome dataset reported here which will be a valuable supplement to the rubber tree genomic and transcriptome information.

Differential gene expression, especially the up-regulated expression of the key genes in the rubber-biosynthesis-related metabolic pathways in response to ethylene allowed us to find the rubber-yield limiting factors. Based on our finding, it is possibly to develop a molecular diagnostic for rubber yield and to improve the rubber tree for high yield through the genetic modification of those key genes [67].

## Conclusions

In the present study, the molecular mechanism behind the ethylene stimulation of rubber yield was revealed. Up-regulation of the key enzymes in the glycolysis pathway and the C3 carbon fixation may be responsible for enhancing the rubber production by ethephon treatment. Identification of the rubber-yield limiting factors possibly leads to development of a molecular approach to assay and predict the productivity of rubber tree, and obtainment of new high yielding cultivars through genetic engineering. Moreover, our transcriptome data provides an useful resources for gene mining for high production of rubber.

## Methods

### Plant material and RNA extraction

Rubber trees of clone PR107 were planted at the Experimental Farm of the Chinese Academy of Tropical Agriculture Science in 2000, and opened for tapping in 1995, on the s/2 d/4 system (half spiral tapped every 4 days) and with 1.5 %-ethephon treatment 1 day before tapping at intervals of 15 days.

Trees of equal size in the same plot were selected and divided into three groups, each with six trees. Ethephon (2-chloroethane phosphonic acid; 1.5 % v/v) was applied using a brush on the tapping panel. Bark samples were collected from two groups by tapping at 8 and 24 h after ethephon application, respectively. Concurrently, bark was collected from a corresponding group of trees at 8 h after treatment in the same way with water only as control. The samples were immediately frozen in liquid nitrogen and shipped on dry ice to BGI Life Tech Co., Ltd (Shenzhen, China) for Illumina sequencing.

Bark RNA was isolated using the TRIzol® Reagent (Invitrogen) following the protocol in the manufacturer’s instructions. RNA integrity was confirmed by a 2100 Bioanalyzer (Agilent Technologies).

### cDNA library construction and sequencing

Three cDNA libraries, C (control), E8 (Ethephon treatment for 8 h) and E24 (Ethephon treatment for 24 h), were generated using the mRNA-Seq 8 sample prep Kit (Illumina) according to the manufacturer’s instructions. Magnetic beads containing poly-T molecules was used to isolate the poly(A) mRNA from 20 μg of total RNA. Following purification, the samples were fragmented into small pieces using divalent cations at 94 °C for 5 min and converted into the first and second-strand cDNA with the SuperScript double-stranded cDNA synthesis kit (Invitrogen, CA). Then, the synthesized cDNA was subject to end repair and adenylation of 3′ ends and purified using the QIAquick PCR Purification Kit (QIAGEN). Afterward, Illumina paired end adapters were ligated to the resulting cDNA fragments. Each cDNA library was finally constructed with an insert size of 200 bp. After quality validating by an Agilent Technologies 2100 Bioanalyzer, deep sequencing was performed with Illumina HiSeqTM 2000 (Illumina Inc., San Diego, CA, USA).

### De novo assembly and gene annotation

Raw reads were filtered by the Illumina pipeline before the assembly. The reads with more than 20 % of bases with a quality value ≤10, unknown nucleotides higher than 5 % and adaptor contamination were removed. Transcriptome de novo assembly was then conducted with short reads assembling program – Trinity (release 20130225; running parameter: seqType fq --min_contig_length 100) (http://trinityrnaseq.github.io/) [68, 69]. The resulting sequences of trinity, termed Unigenes, from each sample’s assembly were taken into further process of sequence splicing and redundancy removing to acquire non-redundant Unigenes as long as possible. Then, the Unigenes were split into two classes: clusters (CL, with some Unigenes which similarity between them was higher than 70 %) and singletons (Unigene). At last, Blast X (v2.2.26 + x64-linux) alignment (an E-value <0.00001; running parameter: -F F -e 1e-5 -p blastx) between the Unigenes and protein databases like NR (release 20130408), SwissProt (release 201303), KEGG (release 63.0) and COG (release 20090331) was carried out. Sequence direction of and functional annotations to the Unigenes were decided and assigned with the best aligning results. The sequence direction of the Unigene unaligned to non of the above databases was determined by a software named ESTScan (v3.0.2) [69]. With the NR annotation, the Blast2GO program (v2.5.0; release 20120801) [70] was used to get the GO annotation for the Unigenes. The GO functional classification for the Unigenes was produced by the WEGO software [71] and the pathway assignments were performed with the help of KEGG database [72].

### Differential gene expression analysis

The normalized expression levels of the Unigenes were calculated using the FPKM method [73]. Then, the identification of DEGs was conducted between C and E8, and C and E24 using a computational method [74] included in SOAPaligner/soap (http://soap.genomics.org.cn/soapaligner.html; v2.21; running parameter: -m 0 -x 500 -s 40 -l 35 -v 5 -r 1), a tool of the Short Oligonucleotide Analysis Package (SOAP) for the RNA-Seq data analysis. The significance of differential transcript abundance was judged with the false discovery rate (FDR) value [75]. Only those DEGs with FDR ≤0.001 with the absolute fold change ≥2 were reserved. The enrichment analysis was performed to find GO terms in which DEGs are significantly enriched comparing to the whole transcriptome, using the hypergeometric test. For the pathway enrichment analysis, all DEGs were mapped to terms in the KEGG database to identify significantly over-represented metabolic pathways or signal transduction pathways. Then the hypergeometric test was performed for the statistical analysis, while the Bonferroni correction was adopted for the multiple testing correction, with a *q*-value cutoff of ≤0.05. From the results, we mainly focused on the differentially regulated pathways closely relevant to the biosynthesis of natural rubber. In order to evaluate expression levels of individual genes in these pathways, we re-mapped the Unigenes to the sequences of each gene and all FPKM values were added together. Then the final FPKM values were used as the expression levels of the individual gene [76]. For these genes, Log2 fold changes were calculated between C and E8, and between C and E24, respectively, while the results were presented and illustrated in the pathways.

Plant Transcription Factor Database (PlantTFDB, v3.0) is a comprehensive database of transcription factors (TFs) in plants, with detailed annotation and classification information [77]. The sequences of all existing TFs were retrieved from PlantTFDB. Then, Blast X search with PlantTFDB was performed and the TFs were identified with an E-value cutoff of ≤1E-5.

### Validation of gene expression by qRT-PCR

To validate our transciptome results, expression of three randomly chosen key genes (*PFK, Rubisco* and *GADPH*) in glycolysis and carbon fixation with significant expression changes in the transcriptome data was verified by qRT-PCR. Total RNA was isolated from the equal amount of bark tissues of three rubber trees of each treatment by following the protocol described by Venkatachalam et al. [78]. The first strand cDNA was synthesized from 2.5 μg of total RNA through a RevertAidTM Premium first strand cDNA synthesis kit (Fermentas). The standard curve for each target gene was obtained by qRT-PCR with series cDNA dilutions of cDNA. The reaction mixture (20 μl) for qRT-PCR comprised of 10 μl SYBR Premix Ex TaqII, 6.8 μl EASY Dilution, 0.4 μl 10 μM Forward primer and 0.4 μl 10 μM Reverse primer. The PCR reactions were performed on an CFX96TM Real-Time PCR Detection System (Bio-Rad) as follows: 95 °C for 30 s, followed by 40 cycles of 95 °C for 5 s, and then annealing at 60–95 °C for 30 s. Expression analysis of each gene was confirmed in three independent reactions with *Actin* gene as an internal control for normalization of the expression levels of the chosen transcripts. The relative expression of the genes was calculated using the 2^−△△Ct^ method. The primer sequences used for qRT-PCR are listed in Table 2.

**Table 2:** The forward and reverse primers used in validation experiment of gene expression by qRT-PCR

Genes	Directions	Sequences
*PFK*	Forward	5′-AATGGCTGGATACACTGGCTTT-3′
	Reverse	5′-AGCCTAGCCCACATCCTATCTG-3′
*Rubisco*	Forward	5′-GTACACAGACCACCAAATGAGCC-3′
	Reverse	5′-TATTCTCAATGCGTTCATCTGCC-3′
*GADPH*	Forward	5′-CCGGTGGTGTTAAATAAGCTTC-3′
	Reverse	5′-ACTGGTCTTCCGTCAATACTCAT-3′
*Actin*	Forward	5′-CAGTGGTCGTACAACTGGTAT-3′
	Reverse	5′-ATCCTCCAATCCAGACACTGT-3′

### Availability of data and materials

The datasets supporting the conclusions of this article are included within the article and its additional files. The clean reads for C, E8 and E24 have been deposited in the National Center for Biotechnology Information (NCBI) Sequence Read Archive (SRA) under accession numbers SRX1134637, SRX1134639 and SRX1134640, respectively. The RNA-seq data in this article has been deposited in the NCBI’s Gene Expression Omnibus (GEO) under accession number GSE78145.

## Additional files

Additional file 1: Summary of functional annotations of unigenes by aligning to the Nr, Nt, Swiss-Prot, COG, GO and KEGG databases. (XLS 24813 kb) Additional file 2: List of DEGs between C and E-8, and C and E-24. (XLS 23067 kb) Additional file 3: The top 20 most up-regulated and down regulated genes between C and E-8, and C and E-24. (XLS 43 kb) Additional file 4: Pathways significantly enriched of DEGs (C vs. E-8, and C vs. E-24). (XLS 196 kb) Additional file 5: DEGs involved in the glycolysis in E8 and E24 compared to C samples of *Hevea brasiliensis*. (XLS 59 kb) Additional file 6: Differential expression of unigenes involved in IPP biosynthesis pathway in E8 and E24 compared to C samples of *Hevea brasiliensis.* AACT: acetoacetyl-CoA thiolase; HMGS: 3-hydroxy-3-metylglutaryl coenzyme A (HMG-CoA) synthase; HMGR: HMG-CoA reductase; MVK: mevalonate (MVA) kinase; PMVK: 5-phosphomevalonate (MVP) kinase; PMD: phosphatemevalonate decarboxylase; DPMD: 5-diphosphomevalonate (MVPP) decarboxylase; IPK: isopentenyl phosphate (IP) kinase; GPS: geranyl diphosphate synthase; IDI: isopentenyl diphosphate (IPP) isomerase; DXS: 1-deoxy-d-xylulose 5-phosphate (DXP) synthase; DXR: DXP reductoisomerase; MCT: 4-(cytidine 5′ -diphospho)-2- C- methyl-D-erythritol (CDP-ME) synthase; CMK: CDP-ME kinase; MDS: 2C-methyl-D-erythritol 2,4-cyclodiphosphate (MEcPP) synthase; HDS: 4-hydroxy-3-methylbut-2-enyl diphosphate (HMBPP) synthase; HDR: HMBPP reductase. Cells with gray border lines in the upper rows represent differentially expressed unigenes in E8 compared to C and cells with green border lines in the lower rows represent differentially expressed unigenes in E24 compared to C. Relative levels of expression are showed by a color gradient from low (blue) to high (red). (JPG 157 kb) Additional file 7: DEGs involved in the IPP biosynthesis in E8 and E24 compared to C samples of *Hevea brasiliensis.* (XLS 31 kb) Additional file 8: DEGs involved in the Calvin cycle in E8 and E24 compared to C samples of *Hevea brasiliensis.* (XLS 43 kb) Additional file 9: Differential expression of unigenes involved in hormone signaling in E8 and E24 compared to C samples of *Hevea brasiliensis.* Ethylene signalling pathway: ETR1: ETHYLENE RESPONSE 1; CTR1: CONSTITUTIVE TRIPLE RESPONSE 1; EIN2: ETHYLENE INSENSITIVE 2; EIN3: ETHYLENE INSENSITIVE 3; ERF1/2: ETHYLENE RESPONSE FACTOR 1/2; EBF1/2: EIN3 binding F-Box protein 1/2; BR signaling pathway: BRI1: Brassinosteroid-Insensitive 1; BAK1: BRI1-associated kinase 1; BKI1: BRI1 KINASE INHIBITOR 1; BSK: BR SIGNALING KINASE; BSU1: bri1 SUPPRESSOR 1; BIN2: BRASSINOSTEROID-INSENSITIVE 2; BZR1/2: BRASSINAZOLE RESISTANT 1/2; TCH: TOUCH genes; CYCD3: CYCLIN D3; GA signaling pathway: GID1: GIBBERELLIN INSENSITIVE DWARF 1; GID2: GIBBERELLIN INSENSITIVE DWARF 2; DELLAs: DELLA growth inhibitors; TF: transcriptional factor; Auxin signaling pathway: AUX1: AUXIN1; TIR1: TRANSPORT INHIBITOR RESPONSE 1; IAA: INDOLE ACETIC ACID; ARF: AUXIN RESPONSE FACTOR; SAUR: Small Auxin-Up RNA; G10H: geraniol 10-hydroxylase gene; Cytokinin signaling pathway: CRE1: CYTOKININ RESPONSE 1; AHP: histidine phosphotransfer protein; B-ARR: type-B response regulator (ARR); A-ARR: type-A response regulator (ARR); SA signalling pathway: NPR1: Non-expressor of pathogenesis-related genes 1; TGA: the bZIP transcription factors; PR1: pathogenesis related protein 1; JA signaling pathway: JAR1: JASMONATES RESISTANT 1; JA-Ile: jasmonoyl isoleucine; JAZ: Jasmonate ZIM-domain-containing protein; MYC2: a basic helix-loop-helix (bHLH) transcription factor; ORCA3: Octadecanoid-derivative Responsive Catharanthus AP2-domain gene; ABA signalling pathway: PYR1/PYLs: Pyrabactin Resistance Protein1/PYR-Like proteins; PP2Cs: protein phosphatases which fall under the category of type 2C; SnRK2: SNF1 (Sucrose-Nonfermenting Kinase1)-related protein kinase 2: ABF: ABA responsive element (ABRE) binding factors. Cells with gray border lines in the upper rows represent differentially expressed unigenes in E8 compared to C and cells with green border lines in the lower rows represent differentially expressed unigenes in E24 compared to C. Relative levels of expression are showed by a color gradient from low (blue) to high (red). (JPG 249 kb) Additional file 10: DEGs of hormone signaling components in E8 and E24 compared to C samples of *Hevea brasiliensis.* (XLS 126 kb) Additional file 11: DEGs of transcription factors in E8 and E24 compared to C samples of *Hevea brasiliensis.* (XLS 256 kb) Additional file 12: The top five up-regulated and down-regulated TFs in E8 and E24 compared to C. (XLS 23 kb)

## Abbreviations

A -ARR: type-A response regulator
ABA: abscisic acid
acetyl-CoA: acetyl-coenzyme A
ACS: acetyl-coenzyme A synthetase or acetate, CoA ligase
ADH: alcohol dehydrogenase
ALDH: aldehyde dehydrogenase
bHLH: the basic helix-loop-helix family
BIN2: brassinosteroid-insensitive 2
1,3-BPG: 1,3-bisphosphoglycerate
BR: brassinosteroid
BZR1/2: brassinazole resistant 1/2
COG: Cluster of Orthologous Groups
DEGs: differentially expressed genes
DEX/MEP: 1-dexoxy-D-xylulose 5-phosphate/2-C-methyl-D-erythritol 4-phosphate
DHAP: dihydroxyacetone phosphate or glycerone phosphate
EBF1/2: EIN3 binding F-Box protein 1/2
EIN3: ethylene insensitive 3
ERF1: ethylene response factor 1
ERF: the ethylene-responsive element binding factor family
ETR: ethylene response
FBA: fructose 1,6-bisphosphate aldolase
FBP: fructose-1,6-bisphosphatase
FDR: false discovery rate
FPKM (RPKM): fragments per kb per million reads
GA: gibberellin
G3P or GA3P: glyceraldehyde 3-phosphate
GAPDH: glyceraldehyde 3-phosphate dehydrogenase
GO: gene ontology
GS: glutamine synthetase
HK: hexokinase
HMGR: 3-hydroxy-3-metylglutaryl coenzyme A reductase
IAA: indole acetic acid
IPP: isopentenyl diphosphate
JA: jasmonate
KEGG: SwissProt, Kyoto Encyclopedia of Genes and Genomes
MVA: mevalonic acid
MYB: v-myb avian myeloblastosis viral oncogene homolog
NCBI: National Center for Biotechnology Information
NR: NCBI’s non-redundant protein databases
NT: non-redundant NCBI nucleotide database
PFK: phosphofructokinase
PGI: phosphoglucoisomerase
PGK: phosphoglycerate kinase
PGM: phosphoglycerate mutase
PEP: phosphoenolpyruvate
2-PG: 2-phosphoglycerate
3-PG: 3-phosphoglycerate
PlantTFDB: Plant Transcription Factor Database
PK: pyruvate kinase
PRK: phosphoribulokinase
RPI: ribose-5-phosphate isomerase
RubisCo: ribulose-1,5-bisphosphate carboxylase/oxygenase
RuBP: ribulose-1,5-bisphosphate
SA: salicylic acid
SBP: sedoheptulose-1,7-bisphosphatase
SOAP: short oligonucleotide analysis package
SRA: sequence read archive
TFs: transcription factors
TK: transketolase
TPI or TIM: triose-phosphate isomerase
