# Supplementary figures and images for: Molecular mechanism of ethylene stimulation of latex yield in rubber tree (Hevea brasiliensis) revealed by de novo sequencing and transcriptome analysis
Source: BMC Genomics. 2016 Mar 24;17:257. doi: 10.1186/s12864-016-2587-4 (PMC4806457; doi:10.1186/s12864-016-2587-4)

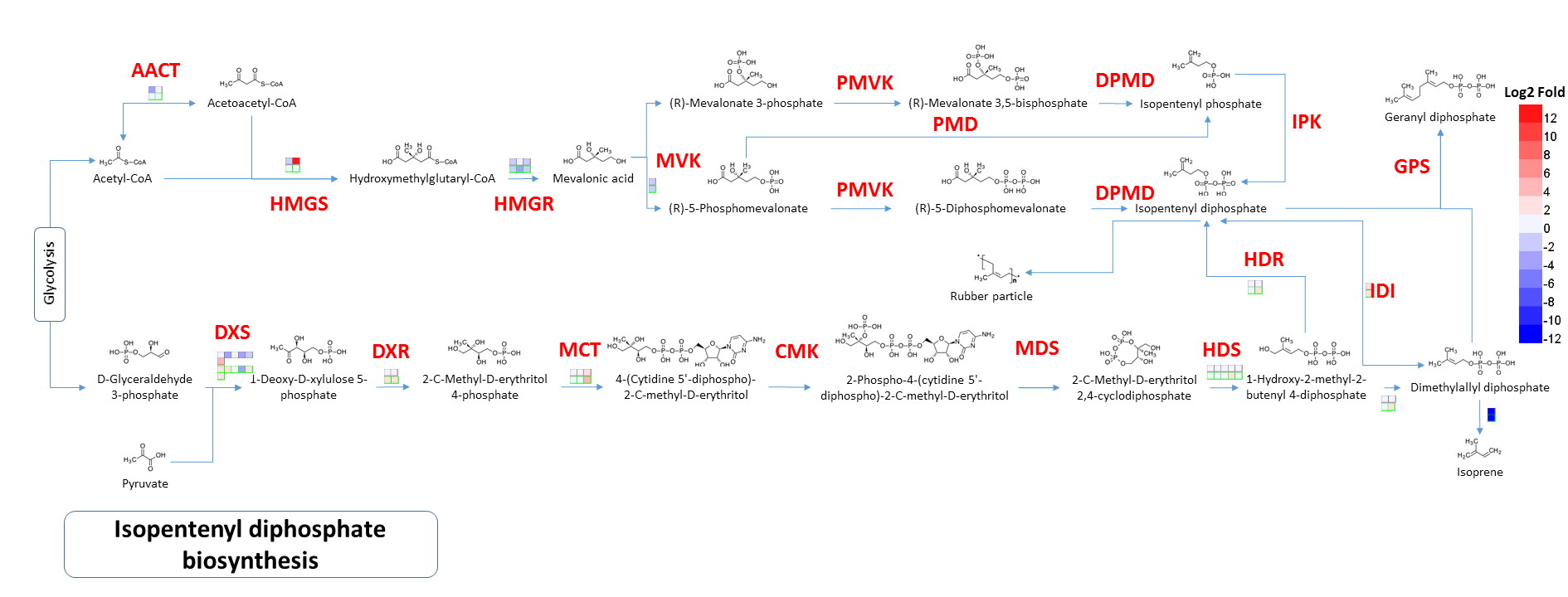

Supplement: Additional file 6: — Differential expression of unigenes involved in IPP biosynthesis pathway in E8 and E24 compared to C samples of Hevea brasiliensis. AACT: acetoacetyl-CoA thiolase; HMGS: 3-hydroxy-3-metylglutaryl coenzyme A (HMG-CoA) synthase; HMGR: HMG-CoA reductase; MVK: mevalonate (MVA) kinase; PMVK: 5-phosphomevalonate (MVP) kinase; PMD: phosphatemevalonate decarboxylase; DPMD: 5-diphosphomevalonate (MVPP) decarboxylase; IPK: isopentenyl phosphate (IP) kinase; GPS: geranyl diphosphate synthase; IDI: isopentenyl diphosphate (IPP) isomerase; DXS: 1-deoxy-d-xylulose 5-phosphate (DXP) synthase; DXR: DXP reductoisomerase; MCT: 4-(cytidine 5′ -diphospho)-2- C- methyl-D-erythritol (CDP-ME) synthase; CMK: CDP-ME kinase; MDS: 2C-methyl-D-erythritol 2,4-cyclodiphosphate (MEcPP) synthase; HDS: 4-hydroxy-3-methylbut-2-enyl diphosphate (HMBPP) synthase; HDR: HMBPP reductase. Cells with gray border lines in the upper rows represent differentially expressed unigenes in E8 compared to C and cells with green border lines in the lower rows represent differentially expressed unigenes in E24 compared to C. Relative levels of expression are showed by a color gradient from low (blue) to high (red). (JPG 157 kb) [file 12864_2016_2587_MOESM6_ESM.jpg]

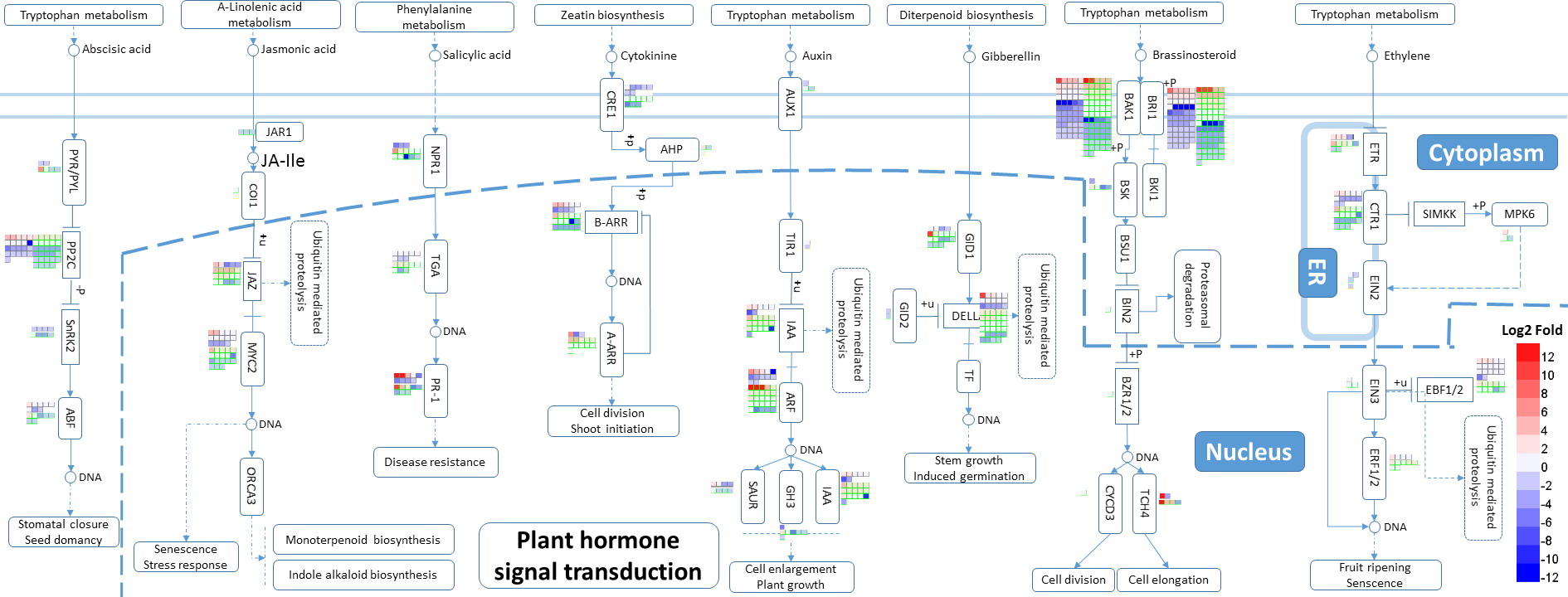

Supplement: Additional file 9: — Differential expression of unigenes involved in hormone signaling in E8 and E24 compared to C samples of Hevea brasiliensis. Ethylene signalling pathway: ETR1: ETHYLENE RESPONSE 1; CTR1: CONSTITUTIVE TRIPLE RESPONSE 1; EIN2: ETHYLENE INSENSITIVE 2; EIN3: ETHYLENE INSENSITIVE 3; ERF1/2: ETHYLENE RESPONSE FACTOR 1/2; EBF1/2: EIN3 binding F-Box protein 1/2; BR signaling pathway: BRI1: Brassinosteroid-Insensitive 1; BAK1: BRI1-associated kinase 1; BKI1: BRI1 KINASE INHIBITOR 1; BSK: BR SIGNALING KINASE; BSU1: bri1 SUPPRESSOR 1; BIN2: BRASSINOSTEROID-INSENSITIVE 2; BZR1/2: BRASSINAZOLE RESISTANT 1/2; TCH: TOUCH genes; CYCD3: CYCLIN D3; GA signaling pathway: GID1: GIBBERELLIN INSENSITIVE DWARF 1; GID2: GIBBERELLIN INSENSITIVE DWARF 2; DELLAs: DELLA growth inhibitors; TF: transcriptional factor; Auxin signaling pathway: AUX1: AUXIN1; TIR1: TRANSPORT INHIBITOR RESPONSE 1; IAA: INDOLE ACETIC ACID; ARF: AUXIN RESPONSE FACTOR; SAUR: Small Auxin-Up RNA; G10H: geraniol 10-hydroxylase gene; Cytokinin signaling pathway: CRE1: CYTOKININ RESPONSE 1; AHP: histidine phosphotransfer protein; B-ARR: type-B response regulator (ARR); A-ARR: type-A response regulator (ARR); SA signalling pathway: NPR1: Non-expressor of pathogenesis-related genes 1; TGA: the bZIP transcription factors; PR1: pathogenesis related protein 1; JA signaling pathway: JAR1: JASMONATES RESISTANT 1; JA-Ile: jasmonoyl isoleucine; JAZ: Jasmonate ZIM-domain-containing protein; MYC2: a basic helix-loop-helix (bHLH) transcription factor; ORCA3: Octadecanoid-derivative Responsive Catharanthus AP2-domain gene; ABA signalling pathway: PYR1/PYLs: Pyrabactin Resistance Protein1/PYR-Like proteins; PP2Cs: protein phosphatases which fall under the category of type 2C; SnRK2: SNF1 (Sucrose-Nonfermenting Kinase1)-related protein kinase 2: ABF: ABA responsive element (ABRE) binding factors. Cells with gray border lines in the upper rows represent differentially expressed unigenes in E8 compared to C and cells with green border lines [file 12864_2016_2587_MOESM9_ESM.jpg]
